# Supplementary material for: Causal Relationship between Aging and Anorexia Nervosa: A White-Matter-Microstructure-Mediated Mendelian Randomization Analysis
Source: Biomedicines. 2024 Aug 16;12(8):1874. doi: 10.3390/biomedicines12081874 (PMC11351342; doi:10.3390/biomedicines12081874)
Supplement: Supplementary file 1 [file biomedicines-12-01874-s001.zip › Supplementary Figure/Figure S4/Singe SNP plot of MR analysis for TL to ubm-b-1851.pdf]

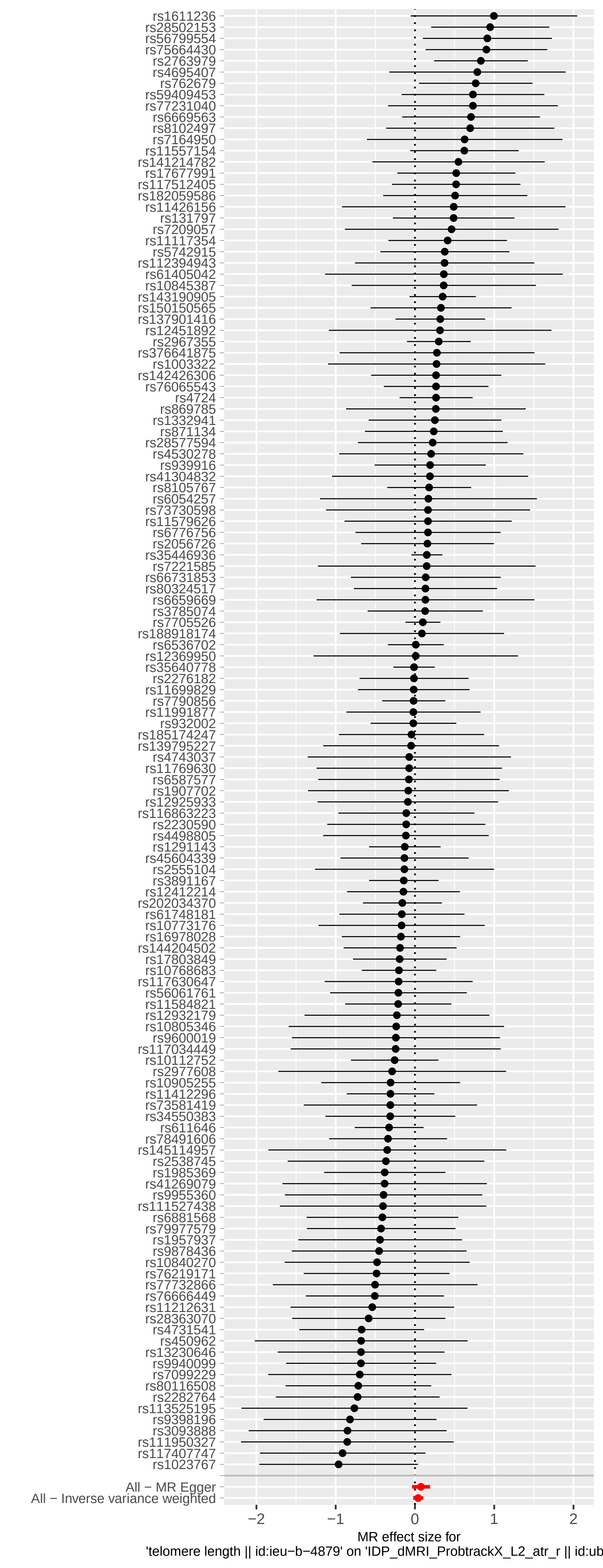
rs1611236
rs28502153
rs56799554
rs75664430
rs2763979
rs4695407
rs762679
rs59409453
rs77231040
rs6669563
rs8102497
rs7164950
rs11557154
rs141214782
rs17677991
rs117512405
rs182059586
rs11426156
rs131797
rs7209057
rs11117354
rs5742915
rs112394943
rs61405042
rs10845387
rs143190905
rs150150565
rs137901416
rs12451892
rs2967355
rs376641875
rs1003322
rs142426306
rs76065543
rs4724
rs869785
rs1332941
rs871134
rs28577594
rs4530278
rs939916
rs41304832
rs8105767
rs6054257
rs73730598
rs11579626
rs6776756
rs2056726
rs35446936
rs7221585
rs66731853
rs80324517
rs6659669
rs3785074
rs7705526
rs188918174
rs6536702
rs12369950
rs35640778
rs2276182
rs11699829
rs7790856
rs11991877
rs932002
rs185174247
rs139795227
rs4743037
rs11769630
rs6587577
rs1907702
rs12925933
rs116863223
rs2230590
rs4498805
rs1291143
rs45604339
rs2555104
rs3891167
rs12412214
rs202034370
rs61748181
rs10773176
rs16978028
rs144204502
rs17803849
rs10768683
rs117630647
rs56061761
rs11584821
rs12932179
rs10805346
rs9600019
rs117034449
rs10112752
rs2977608
rs10905255
rs11412296
rs73581419
rs34550383
rs611646
rs78491606
rs145114957
rs2538745
rs1985369
rs41269079
rs9955360
rs111527438
rs6881568
rs79977579
rs1957937
rs9878436
rs10840270
rs76219171
rs77732866
rs76666449
rs11212631
rs28363070
rs4731541
rs450962
rs13230646
rs9940099
rs7099229
rs80116508
rs2282764
rs113525195
rs9398196
rs3093888
rs111950327
rs117407747
rs1023767
All − MR Egger
All − Inverse variance weighted
−2
−1
0
1
2
MR effect size for
'telomere length || id:ieu−b−4879' on 'IDP_dMRI_ProbtrackX_L2_atr_r || id:ub
